# Properties of *Astragalus* sp. microsymbionts and their putative role in plant growth promotion

**DOI:** 10.1007/s00203-016-1243-3

**Published:** 2016-05-21

**Authors:** Sylwia Wdowiak-Wróbel, Wanda Małek

**Affiliations:** Department of Genetics and Microbiology, Maria Curie Skłodowska University, Akademicka 19 St., 20-033 Lublin, Poland

**Keywords:** *Astragalus* sp. microsymbionts, *acdS* gene, PGPR

## Abstract

The plant growth-promoting rhizobacteria have developed many different (indirect and direct) mechanisms that have a positive effect on plant growth and development. Strains isolated from *Astragaluscicer* and *Astragalusglycyphyllos* root nodules were investigated for their plant growth-promoting properties such as production of indole-3-acetic acid (IAA) and siderophores, phosphate solubilization, ACC deaminase activity, and tolerance to heavy metals. IAA production and P-solubilization were frequent features in the analysed strains, while siderophores were not produced by any of them. In this work, we investigated the presence of the *acdS* genes and ACC deaminase activities in *Astragalauscicer* and *A*. *glycyphyllos* microsymbionts, classified within the genus *Mesorhizobium*. The results demonstrated that the *acdS* gene is widespread in the genome of *Astragalus* sp. microsymbionts; however, none of the tested strains showed ACC deaminase activity. The *acdS* gene sequence similarity of the analysed strains to each other was in the range from 84 to 99 %. On the phylogram of *acdS* gene sequences of milkvetch, the symbionts clustered tightly with the genus *Mesorhizobium* bacteria.

**Electronic supplementary material:**

The online version of this article (doi:10.1007/s00203-016-1243-3) contains supplementary material, which is available to authorized users.

## Introduction

Ethylene affects plant growth and development. It is responsible for several processes in plants and, depending on the level, can e.g. promote root initiation, inhibit root elongation, activate plant hormone synthesis, and promote flower wilting. Ethylene is also involved in the response to both biotic and abiotic stresses. An increase in ethylene synthesis may accompany for example extreme temperatures, water flooding, drought, radiation, salinity, and presence of various pathogens. It has also been described that ethylene affects various stages of symbiosis (Vacheron et al. 2013; Glick 2014). Ethylene can inhibit nodule development in different fabacean plants, for example in *Phaseolusvulgaris*, *Lotusjaponicas*, and *Trifoliumrepens* (Tamimi and Timko 2003).

In the literature, there is a “stress ethylene” concept. The model of “stress ethylene” includes the synthesis of ethylene in two peaks. The first one is small and reflects ethylene that consumes the pool of ACC (1-aminocyclopropane-1-carboxylate) existing in stressed plant tissues. Probably, this ethylene is responsible for initiation of transcription of genes whose products are involved in defensive or protective mechanisms. The second, bigger ethylene peak reflects synthesis of additional ACC in plant response to stress. It is responsible for initiation of such processes as cell death, senescence, and chlorosis (Glick 2014; Singh et al. 2015).

Plant growth promotion by rhizosphere microorganisms (plant growth-promoting rhizobacteria, PGPR) is a result of various mechanisms such as production of indole-3-acetic acid (IAA), siderophores, 2,3-butanediol, lytic enzymes, and 1-aminocyclopropane-1-carboxylate deaminase (ACC deaminase) as well as induction of plant systemic resistance to pathogens. It has been demonstrated that inoculation of plants with ACC deaminase-producing rhizobia causes a decrease in the plant ethylene level, which in turn protects plant from the effects of biotic and abiotic stresses (Saleem et al. 2007; Bhattacharyya and Jha 2012; Beneduzi et al. 2012).

ACC deaminase (EC:4.1.99.4) synthesized by microorganisms converts ACC, the immediate precursor of ethylene in plants, into ammonia and ketobutyrate, which can be used as a source of nitrogen and carbon, respectively. This enzyme synthesized by soil bacteria decreases the level of ethylene in plants and, in consequence, stimulates plant growth. A low level of ethylene correlates with higher resistance of plants to various kinds of biotic and abiotic stresses such as high salt, extreme temperature, phytopathogenic infection. The presence of ACC deaminase-producing microorganisms in soil contributes to longer plant roots and shoots and to higher plant resistance to inhibition of growth by ethylene stress (Bhattacharyya and Jha 2012; Glick 2014).

It is worth noting that, under stress conditions, some microorganisms produce the phytohormone indole-3-acetic acid (IAA). IAA is a natural plant auxin that is the common product of l-tryptophan metabolism. The indole-3-acetic acid enhances development of longer roots with an increased number of root hairs and lateral roots. IAA inhibits or delays leaf abscission and affects plant flowering and fruiting. This auxine may stimulate such processes as tissue differentiation, xylem formation, nitrogen fixation, and plant stress resistance. It has been found that production of IAA by bacteria can promote plant growth and increase *acdS* gene transcription (Zaidi et al. 2010; Zhao 2010). The application of microorganisms producing both IAA and ACC deaminase as plants inoculates did not result in any increase of the ethylene level in contrast to plants inoculated only with IAA-producing bacteria (Glick 2014).

PGPR are able to synthesize different compounds that have a positive impact on the growth, development, and tolerance to different stresses in plants. These effects are related, inter alia, to nutrient enrichment of soil by phosphate solubilization, nitrogen fixation, or lipase and protease production. PGP microorganisms are often characterized by tolerance to abiotic stresses such as pH, drought, salinity, and heavy metal pollution (Zaidi et al. 2010; Zhao 2010; Beneduzi et al. 2012; Ahemad and Kibret 2014).

We have studied eight mesorhizobium strains isolated from nodules of *Astragaluscicer* and *Astragallusglycyphyllos* growing in Poland, Ukraine, and Canada (Wdowiak and Małek 2000; Gnat et al. 2014). In our earlier studies, *A*. *glycyphyllos* and *A*. *cicer* symbionts were classified into the genus *Mesorhizobium*, based on sequence analysis of 16S rRNA and housekeeping genes (Wdowiak-Wróbel and Malek 2010; Gnat et al. 2014, 2015a). Additionaly *A*. *glycyphyllos* nodule isolates were affiliated into the *M*. *amorphae* and *M*. *ciceri* species by DNA/DNA hybridization and to the new symbiovar, *glycyphyllae*” using *nodA* and *nodC* sequence analysis (Gnat et al. 2015a, b). The strains, representing different phenotypic and genomic groups of *A*. *glycyphyllos* and *A*. *cicer* isolates, were used to determine the ability to use ACC as a sole nitrogen source, the presence of the *acdS* gene in their genome, phylogeny of the *acdS* genes, tolerance of bacteria to heavy metals, and their capability of IAA synthesis and phosphate solubilization.

## Materials and methods

### Bacterial strains

Mesorhizobium strains ACMP18, USDA 3350, AW1/3, and CIAM0210 isolated from *A*. *cicer* and AG1, AG15, AG17, and AG27 isolated from root nodules of *A*. *glycyphyllos*, i.e. two fabacean plant species, were used in this study. Mannitol-yeast extract liquid medium YEM and mannitol-yeast extract agar YEMA were routinely used for culturing and maintenance of the rhizobia (Vincent 1970). The analysed rhizobia were maintained on the YEMA medium at 4 °C.

### Phosphate solubilization

The phosphate solubilizing ability of the rhizobia was tested on Pikovskaya’s agar medium (Pikovskaya 1948). After 7 days of growth at 28 °C, bacteria that induced a clear zone around the colonies were considered to be positive for phosphate solubilization. The capability of the bacteria of phosphate solubilization was described by the solubilization index = the ratio of the total diameter (colony + halo zone) to the colony diameter (Edi-Premono et al. 1996).

### IAA production

*A*. *cicer* and *A*. *glycyphyllos* microsymbionts were screened for their ability to produce IAA. The isolates were grown in Tris-TMRT medium (Manassila et al. 2007) and incubated at 28 °C for 5 days. The presence of IAA was estimated by adding 2 ml of Salkowski’s reagent (2 % 0.5 FeCl_3_ in 35 % HClO solution) into the bacterial culture and incubation of the mixture in the dark at 28 °C for 30 min. IAA concentrations ranging from 10 to 100 µg/ml were used as a positive control.

### Detection of siderophores

Chrome-Azurol S (CAS) agar medium devoid of iron was used for detection of siderophores (Schwyn and Neilands 1987). The bacteria were grown in the synthetic medium described by Jadhav and Desai (1992) with and without 10 μM iron for 24 h on a rotary shaker at 28 ± 2 °C. Next, the cultures were centrifuged and the cell free supernatant was dropped onto CAS plates and incubated in the dark at 28 °C for 4–5 days. The blue colour of the CAS medium is due to the dye complexed with iron. In the presence of the siderophore, the ferric ions are bound, releasing a free dye, which is orange in colour (positive reaction).

### Zn, Cd, Pb

The tolerance of *A*. *cicer* and *A*. *glycyphyllos* symbionts to heavy metals was investigated on yeast mannitol agar (YMA) medium supplemented with various soluble heavy metal salts, namely Cd, Pb, and Zn, at different concentrations. Pb was applied as Pb(CH_3_COO)_2_ (500 and 750 μg ml^−1^), Cd as CdSO_4_ × 8H_2_O (50 and 100 μg ml^−1^), and Zn as ZnSO_4_ × 7H_2_O (250, 500 and 750 μg ml^−1^). Resistance of the *Astragalus* sp. microsymbionts to heavy metals was determined by their growth on plates incubated at 28 °C for 4–5 days.

### Ability to utilize ACC as a sole source of nitrogen

The analysed mesorhizobia were screened for their ability to utilize ACC as a sole nitrogen source in microtiter plates according to the method described by Shahzad et al. (2010).

### ACC deaminase activity

To determine ACC deaminase activity, *A*. *cicer* and *A*. *glycyphyllos* symbionts were grown in 5 ml of TY medium (Beringer 1974) at 30 °C for 2–3 days until they reached the stationary phase. The bacterial cells were centrifuged and washed twice with 0.1 M Tris–HCl (pH 7.5). Next, the bacteria were suspended in 2 ml of M9 minimal medium supplemented with ACC (final concentration of 5 mM) and incubated at 30 °C with shaking for 36 h. ACC deaminase activity was determined according to the method described before (Ma et al. 2003; Penrose and Glick 2003). ACC deaminase activity was determined by measuring the production of α-ketobutyrate (Honma and Shimomura 1978).

The protein concentration in the cell extracts was determined by the method of Bradford (1976) using the Bio-Rad protein reagent (Bio-Rad; Protein Assay Dye Reagent Concentrate #500-0006).

### PCR amplification and sequencing

The bacterial strains were grown in 5 ml of YEM liquid medium at 28 °C for 2–3 days until they reached the stationary phase. Next, the rhizobia were centrifuged at 20,000×*g* for 10 min. Genomic DNA was isolated from the rhizobial strains using the GES method (Pitcher et al. 1989).

To obtain the *acdS* gene sequences, the following set of degenerate primers was designed: primers *acdSF* (5′CAAGCTGCGCAAGCTCGAATA3′) and *acdSR* (5′CATCCCTTGCATCGATTTGC3′). The PCR assay was performed according to the manufacturer’s description using 25-μl of a reaction mixture (Sigma) under the following conditions: initial denaturation for 5 min at 95 °C, followed by 35 cycles of 30 s at 95 °C, 30 s at 50 °C, and 1 min at 72 °C, and final 5 min elongation at 72 °C. The PCR products were purified using a Clean-up kit (A&A Biotechnology) and sequencing reactions were performed using the BigDye Terminator Cycle Sequencing Kit (Applied Biosystems, USA). The products obtained were cleaned with an Ex-Terminator kit (A&A Biotechnology) and analysed in an automatic 3500 Genetic Analyzer sequencer (Applied Biosystems). The sequences of the *acdS* genes were compared with the sequences available in the GenBank and aligned using ClustalX2 multiple sequence alignment (Larkin et al. 2007). The phylogenetic tree of the *acdS* and AcdS sequences were constructed by MEGA 4.0 software (Tamura et al. 2007). The sequence similarity rate of the *acdS* sequence genes was determined according to the Kimura’s two-parameter model (Kimura 1980). The phylogenetic tree of the *acdS* gene sequences and deduced AcdS sequences were constructed using the neighbour-joining (NJ) method (Saitou and Nei 1987).

### Labelling the probe with digoxigenin

The purified PCR product was tagged with digoxigenin (DIG) using a DIG Oligonucleotide 3′–end labelling kit (Roche Diagnostics GmbH, Mannheim, Germany). The reactions were carried out in a 50-μl reaction mixture containing 50 ng of template (DNA of *Mesorhizobiumhuakuii* MAFF303099), 5 μl reaction buffer (1×), 5 μl PCR DIG labelling Mix (200 μM dNTP), upstream and downstream primer (30 pmol each), and 0.75 μl enzyme mix (2.6 U). PCR was conducted in a DNA 2720 Thermal Cycler (Applied Biosystems) under the following conditions: 2 min initial denaturation at 94 °C, 10 cycles of 30 s denaturation at 95 °C, annealing at 60 °C for 30 s and 40 s of elongation at 72 °C, 20 cycles of 30 s denaturation at 95 °C, annealing at 60 °C for 30 s and 40 s plus 20 s for each successive cycle of elongation at 72 °C, followed by final extension at 72 °C for 7 min. The presence of the PCR labelling product (~710 bp) was checked by electrophoresis on 1 % agarose gel.

### Preparation of the genomic DNA for Southern hybridization

Approximately 2 μg of genomic DNA isolated from each rhizobial strain was completely digested overnight with 10 U restriction enzyme *Hind*III (MBI Fermentas, Inc.) at 37 °C. The digested DNA was loaded and run on a 1 % agarose gel. 1-kb DNA ladder (MBI Fermentas, Inc.) was used as a molecular weight standard. *Hind*III digested genomic DNA of *M*. *huakuii* MAFF303099 was used as a positive control of the presence of the *acdS* gene. The DNAs were transferred onto a nylon membrane according to Sambrook and Russell (2001). The blot was hybridized with the *acdS* probe. Hybridization was performed according to the procedure described by the manufacturer (DIG Luminescent Detection Kit, Roche).

### Accession numbers

The *acdS* sequences of the milkvetch microsymbionts studied in this work have been deposited in the GenBank database under accession numbers KU745724-KU745731.

## Results

### Growth of *A*. *cicer* and *A*. *glycyphyllos* microsymbionts on ACC medium as a sole nitrogen source

Four symbionts of *A*. *cicer* and four strains isolated from root nodules of *A*. *glycyphyllos* were tested for the capacity of utilization of ACC as a sole N source. The studied milkvetch microsymbionts showed good growth on the control medium-(NH_4_)_2_SO_4_ and much weaker growth on the ACC medium even after 96 h. The ability to use ACC as a sole nitrogen source suggests that the strains isolated from *Astragalus* sp. plants can possess ACC deaminase, i.e. an ACC-hydrolysing enzyme.

### Southern analysis

The Southern hybridization was used to determine the number of *acdS* gene copies in each investigated mesorhizobium strain. The DNAs of the milkvetch microsymbionts were digested with the *Hind*III enzyme, which does not possess restriction sites within the amplified *acdS* genes. Then, restriction fragments were separated by agarose gel electrophoresis and blotted onto Hybond-N+. The blot was hybridized with a *M*. *huakuii* MAFF303099 strain *acdS* gene probe.

In the microsymbionts of the *Astragalus* sp., the copy of the *acdS* gene encoding ACC deaminase was detected using the Southern hybridization method. The results showed that strains AG1, AG15, AG17 and AG27 had a single band with the size of ca. 8000 bp. Strains ACMP18, USDA3350, CIAM0210, and AW1/3 also showed a single band, whose sizes were in the range of ca. 2500, 2200, 4000, and 4000, respectively.

### Analysis of nucleotide and protein sequences of *acdS* genes of *A*. *cicer* and *A*. *glycyphyllos* symbionts

Phylogenetic analysis of the housekeeping genes of astragali isolates determined in the earlier studies indicated that the analysed isolates represent the genus *Mesorhizobium* (Wdowiak-Wróbel and Małek 2010; Gnat et al. 2015a, b). It was found that many bacteria of the genus *Mesorhizobium* have the ACC deaminase-coding *acdS* gene, which plays an important role in the growth and nodulation of fabacean legume plants (Ma et al. 2003, 2004; Glick et al. 2007; Conforte et al. 2010; Nascimento et al. 2012a, b).

**Fig. 1:**
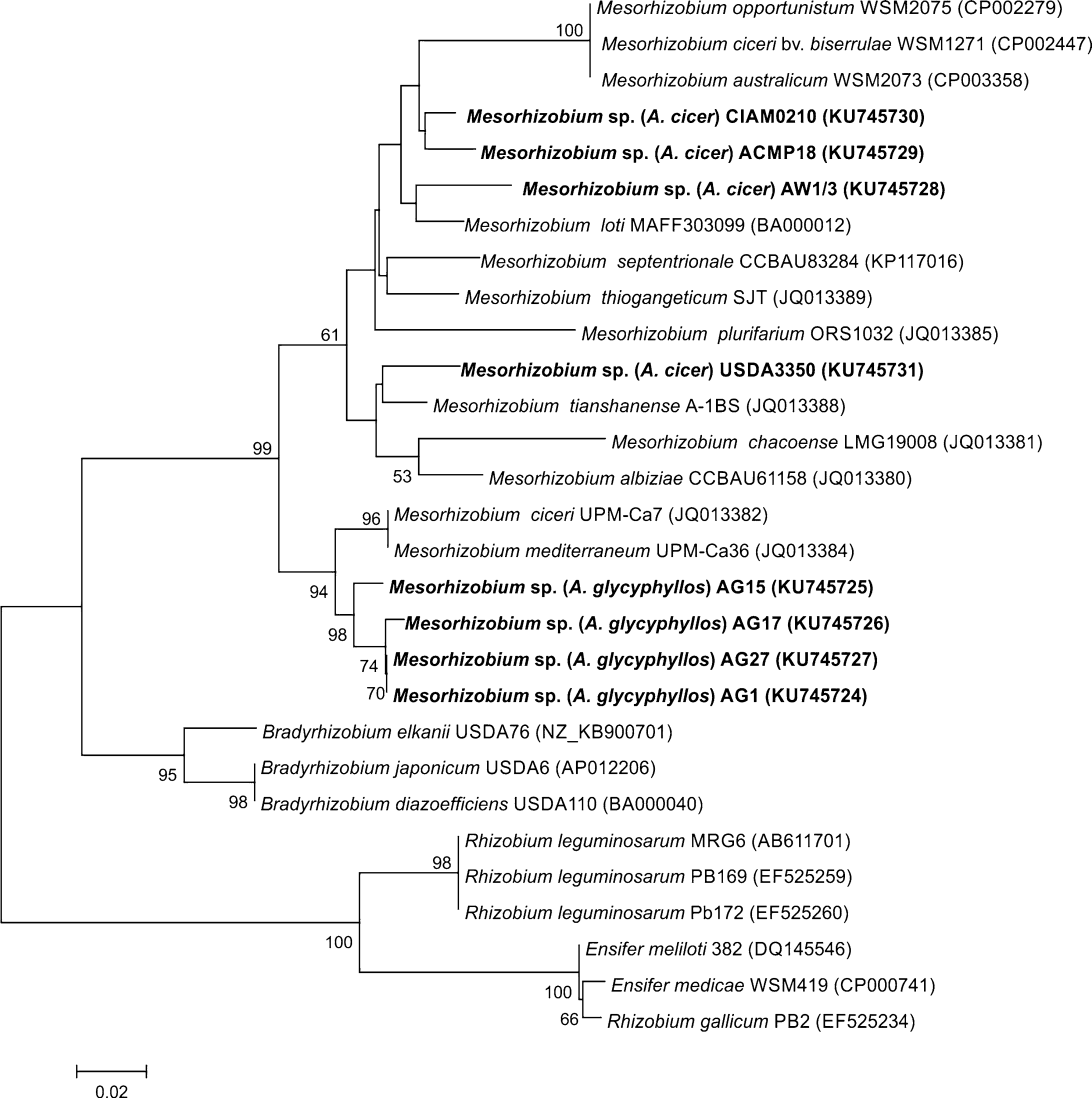
Phylogenetic relationship between *A*. *cicer* and *A*. *glycyphyllos* microsymbionts and reference strains based on partial *acdS* sequences. Bootstrap values (1000 replicates) are shown when higher than 50 %. The *scale bar* represents the percentage of substitutions per site

Although the astragali symbionts did not show ACC deaminase activity in vitro, the *acdS* gene sequences were obtained for all the analysed strains. The phylogenetic analysis of the aligned 529-bp *acdS* gene sequences resulted in the tree shown in Fig. 1. In the phylogenetic tree of partial *acdS* gene sequences, the *A*. *cicer* and *A*. *glycyphyllos* strains were split into 3 main well supported clusters and all of them grouped together with the genus *Mesorhizobium* strains within one monophyletic group (99 % bootstrap support). The phylogenetic analysis of the *acdS* genes revealed that *A*. *glycyphyllos* microsymbionts showed the greatest similarity to the sequences of the *M*. *ciceri* and *M*. *mediterraneum* strains (93–94 % sequence similarity), forming a strongly (99 % bootstrap) supported subgroup with these bacteria (Fig. 1). The sequences of the *acdS* genes of *A*. *cicer* microsymbionts AW1/3, ACMP18, CIAM0210 were related to the *acdS* of *M*. *loti* (with 89–90 % similarity). The phylogenetic analysis of the *acdS* gene of the USDA3350 strain used in this study showed that it belongs to a common group together with *M*. *chacoense*, *M*. *albiziae*, and *M*. *tianshanense* strains (86, 89, and 90 %, respectively).

The next cluster grouped bacteria of the genera *Rhizobium* and *Ensifer*. The last one comprised bacteria of the genus *Bradyrhizobium*. The high bootstrap values of both these groups showed robustness (100 and 95 %, respectively).

The phylogenetic tree derived from the AcdS protein sequences deduced from the *acdS* genes constructed by the neighbour-joining method is shown in Fig. S1. Only minor topological differences were found when the *acdS* nucleotide sequence tree and the tree based on AcdS amino acid sequences were compared. At the amino acid level, the AcdS protein sequences of *A*.*cicer* and *A*. *glycyphyllos* symbionts were 87–100 % identical to each other and 84–95 % to the AcdS proteins of reference *Mesorhizobium* strains.

### Heavy metals

Heavy metal resistance patterns of *Astragalus* sp. microsymbionts were investigated using three heavy metals, i.e. zinc, cadmium, and lead at the concentration specified in the materials and methods. All isolates were found to be resistant to a 500 µg/ml concentration of Zn and Pb. Moreover, approximately 62 and 87 % of the analysed bacteria were found to be tolerant to 750 µg/ml of Zn and Pb, respectively (Table 1). Assessment of the tolerance of the *A*. *cicer* and *A*. *glycyphyllos* symbionts to cadmium revealed that they were much more sensitive to this heavy metal. They grew in the presence of 50 µg Cd in 1 ml medium but their growth was almost completely inhibited by 100 μg ml^−1^ of Cd in the medium. In the presence of 100 µg of Cd per 1 ml of medium, only two of the eight strains analysed showed weak growth. The inhibitory effect of heavy metals on the growth of the astragali symbionts was the following (in an increasing order): Pb(CH_3_COO)_2_, ZnSO_4_ × 7H_2_O, and CdSO_4_ × 8H_2_O.

**Table 1:** Plant growth-promoting features of *A*. *cicer* and *A*. *glycyphyllos* microsymbionts

Strain	P-solubilization	IAA production	Siderophore production	Tolerant to Zn		Tolerant to Cd		Tolerant to Pb	
				500 μg ml^−1^	750 μg ml^−1^	50 μg ml^−1^	100 μg ml^−1^	500 μg ml^−1^	750 μg ml^−1^
ACMP18	+	+	−	+	−	+	−	+	−
AW1/3	+	−	−	+	+	+	−	+	+
CIAM0210	+	+	−	+	−	+	−	+	+
USD3350	+	+	−	+	−	+	−	+	+
AG1	+	+	−	+	+	+	+/−	+	+
AG15	+	+	−	+	+	+	−	+	+
AG17	+	+	−	+	+	+	−	+	+
AG27	+	+	−	+	+	+	+/−	+	+

+ positive reaction; ± weak reaction; − negative reaction

### Characterization of *A*. *cicer* and *A*. *glycyphyllos* rhizobia for phosphate solubilization ability, and production of IAA and siderophores

All the milkvetch nodulators analysed exhibited phosphate solubilizing activity, which was detected by the formation of a clear halo around bacterial colonies growing on Pitkovskay’s medium (Table 1). The isolates showed a varying P-solubilizing index, i.e. from 1.45 to 2.87. The highest P-solubilization index was shown by strains USDA3350 and ACMP18, i.e. the *A*.*cicer* symbionts, and the lowest index was noted for *A*. *glycyphyllos* isolate AG15.

The microsymbionts of *A*. *cicer* (3 strains) and *A*. *glycyphyllos* (4 strains) showed positive reaction in the test for IAA production. The strongest pink colour, i.e. the highest IAA concentration, was detected in the case of the CIAM0210 strain, while the AW1/3 strain did not synthesize IAA. No ability to produce siderophores was detected in any strains tested (Table 1).

## Discussion

Some soil bacterial strains have a positive impact on plant growth and development. Such bacteria are called plant growth-promoting rhizobia (PGPR) (van Peer and Schippers 1989; Frommel et al. 1991; Kloepper et al. 1988). The positive effect of microorganisms on plant growth is related, inter alia, to protection of plants against phytopathogenic organisms or bacterial synthesis of compounds that improve plant growth and development (Glick 1995; Glick et al. 1999). PGPR can synthesize, e.g. phytohormones (auxin, cytokinins), siderophores, and enzymes that can positively influence plant growth and development or are able to solubilize minerals (i.e. phosphorus, potassium, zinc). One of the mechanisms is connected with production of the phytohormone indole-3-acetic acid (IAA). It was shown that IAA synthesized by bacteria participates in plant–microbe signalling and contributes in roots proliferation and elongation (Vessey 2003). It should be noted that bacterial IAA can also stimulate the activity of ACC synthetase, thereby increasing ACC synthesis (Glick 2012). The ability to synthesize IAA has been found in many rhizobia. Studies have shown that this auxin produced by rhizobia affects the nodulation process (Glick et al. 1998; Spaepen and Vanderleyden 2011). Our research showed that 87.5 % of *A*. *cicer* and *A*. *glycyphyllos* microsymbionts were able to produce indole-3-acetic acid. The ability to synthesize IAA has also been described, among others, for *Rhizobiumleguminosarum*, *Mesorhizobiumcicer*, and *Mesorhizobiumloti* strains (Chandra et al. 2007; Ahmad et al. 2008).

Phosphorus is one of the nutrients necessary for plant growth and development. Unfortunately, it occurs in soil mainly in the insoluble forms. Plants can absorb phosphorus only in two soluble forms, i.e. as the monobasic (H_2_PO_4_^−^) and the diabasic (HPO_4_^2−^) ions (Glass 1989). Some PGPR are able to transform the insoluble form of phosphorus to a form available to the plant by acidification of the medium and by phosphorus chelation and transport to the cell (Hameeda et al. 2008; Richardson et al. 2009). It was noticed that inoculation of plants with phosphorus-solubilizing bacteria positively affects the development of the plants (Kucey et al. 1989; Chabot et al. 1996). The ability to solubilize inorganic phosphate has been described in many rhizobial species e.g. *R*. *leguminosarum* bv. *viciae*, *Ensifermeliloti*, *Mesorhizobiummediterraneum*, and *M*. *loti* (Peix et al. 2001; Chandra et al. 2007; Bianco and Defez 2010). Our studies showed that mesorhizobia isolated from the root nodules of *A*. *cicer* and *A*. *glycyphyllos* caused P-solubilization visible as a clear zone around the bacterial colony on Pikovskaya’s medium. It is worth noting that the P-solubilization zone for mesorhizobia isolated from the *A*. *cicer* root nodules was bigger than that for the *A*. *glycyphyllos* symbionts.

The development of rhizobium–fabacean plant symbiosis depends on various environmental factors, inter alia, salinity, pH, and the presence of heavy metals. It is worth noting that the presence of heavy metals at low concentrations is essential for many cellular processes in bacteria. For instance, molybdenum, zinc, and nickel are cofactors of many enzymes, cobalt is the central metal component of the vitamin B12 cofactor, and manganese can be an electron acceptor (Ahemad 2012). It has been shown, however, that heavy metals present in soil at higher concentrations interfere with the metabolism of soil bacteria and can decrease their activity and affect the rhizobium–fabacean plant interaction (Ahemad 2012). Bacteria have developed some mechanisms enabling them to survive in the presence of heavy metals, i.e. precipitation of metals as insoluble salts, efflux of metals from the cells, and chelation of metals (Wani et al. 2009; Zaidi et al. 2012). The mechanism of heavy metal toxicity in respect to rhizobia and rhizobium–fabacean symbiosis is poorly known. Wani et al. (2009) described tolerance of *Mesorhizobium* sp. RC1 and RC4 strains to Cr(VI). These strains exerted a beneficial effect on the development of chickpea growing in Cr-contaminated soil (Wani et al. 2009). Vidal et al. (2009) described highly metal-resistant *Mesorhizobium metallidurans* strains, symbionts of *Anthyllisvulneraria*, which tolerated even 16–32 mM Zn and 0.3–0.5 mM Cd in YEM liquid medium (Vidal et al. 2009). A majority of the strains isolated from *A*. *cicer* and *A*.*glycyphyllos* root nodules tolerated much lower concentrations of heavy metals, i.e. 0.65 mM Zn, 0.14 mM Cd, and 2.3 mM Pb. A similar resistance level to Zn (0.05–0.5 mM) and Cd (0.05 mM) was described by Vidal et al. (2009) in the case of *M*. *tianshanense* ORS 2640^T^ and *M*. *mediterraneum* ORS 2739^T^.

The rhizobium–fabacean symbiosis is a complicated process regulated by both partners of this interaction. It is known that the plant hormone ethylene can inhibit rhizobial infection and nodule formation and limit the number of nodules. Some rhizobia are able to reduce “ethylene stress” by 1-aminocyclopropane-1-carboxylic acid (ACC) deaminase synthesis. ACC deaminase breaks down ACC, an ethylene precursor, into ammonium and α-ketobutyrate. It was shown that bacteria producing ACC deaminase protect plants against various environmental stress factors and phytopathogens, delay plant senescence, and affect the nodulation process (Glick 2014; Nascimento et al. 2012a). The presence of the *acdS* gene encoding ACC deaminase has been described in many Gram-positive, i.e. *Bacilluspumilus*, *Mycobacterium* sp., and *Rodococcus* sp., and Gram-negative bacteria, e.g. rhizobial species such as *R*. *leguminosarum* bv. *viciae*, *E*. *meliloti*, *Ensifer medicae*, *Bradyrhizobiumjaponicum*, *M*. *loti*, and *M*. *ciceri* bv. *biserrulae* (Belimov et al. 2001, 2005; Sullivan et al. 2002; Madhaiyan et al. 2006; Murset et al. 2012; Nascimento et al. 2012a, b; Singh et al. 2015). Some investigations have also shown that many bacteria of the genus *Rhizobium* produce ACC deaminase under free-living conditions, whereas the free-living bacteria of the genus *Mesorhizobium* do not exhibit ACC deaminase activity (Nascimento et al. 2012a, b). In mesorhizobia, the activity of ACC deaminase can be observed during their symbiosis with the plant host, as shown in the case of *M*. *huakuii* bv. *loti* MAFF303099 (Uchiumi et al. 2004; Nukui et al. 2006). In this study, we identified *acdS* genes in the analysed *A*. *cicer* and *A*. *glycyphyllos* microsymbionts. The presence of a single copy of the *acdS* gene in the astragali strains was confirmed by Southern hybridization with the *acdS* gene of *M*. *loti* MAFF303099 as a hybridization probe. As in the case of the mesorhizobia studied by Nascimento et al. (2012a, b), no ACC deaminase activity was detected in the free-living *A*. *cicer* and *A*. *glycyphyllos* microsymbionts. It is also worth noting that the rhizobia display a low level of ACC deaminase activity in comparison with free-living and endophytic plant growth-promoting bacteria (Glick 2014). The induction of ACC deaminase activity in bacteria is a complex and relatively slow process. It was demonstrated that expression of this enzyme depends on the presence of ACC but some other amino acids such as l-alanine, DL-alanine, and d-serine are also capable to induce this process. It was also found that abiotic and biotic stress can also induce ACC deaminase activity (Glick 2014; Singh et al. 2015). This indicates that different factors may induce and affect the ACC deaminase activity.

The presence of the *acdS* genes in the genomes of *A*. *cicer* and *A*. *glycyphyllos* microsymbionts as well as the capability of these bacteria of P-solubilization, utilization of 1-aminocyclopropane-1-carboxylic acid (ACC) as a sole N source, and production of phytohormone IAA indicate that the analysed studied rhizobia can be treated as plant growth-promoting rhizobia. Further studies are needed to prove this assumption, especially these concerning the impact of these bacteria on plant growth and expression of astragali *acdS* genes during their symbiosis with fabaceans.

## Electronic supplementary material

Below is the link to the electronic supplementary material.

Fig. S1 Phylogenetic relationship between *A*. *cicer and A*. *glycyphyllos and reference strains* based on deduced AcdS sequences. Bootstrap values (1000 replicates) are shown when higher than 50%. The scale bar represents the percentage of substitutions per site (JPEG 385 kb)
