# Supplementary figures and images for: Properties of Astragalus sp. microsymbionts and their putative role in plant growth promotion
Source: Arch Microbiol. 2016 May 21;198(8):793–801. doi: 10.1007/s00203-016-1243-3 (PMC4995237; doi:10.1007/s00203-016-1243-3)

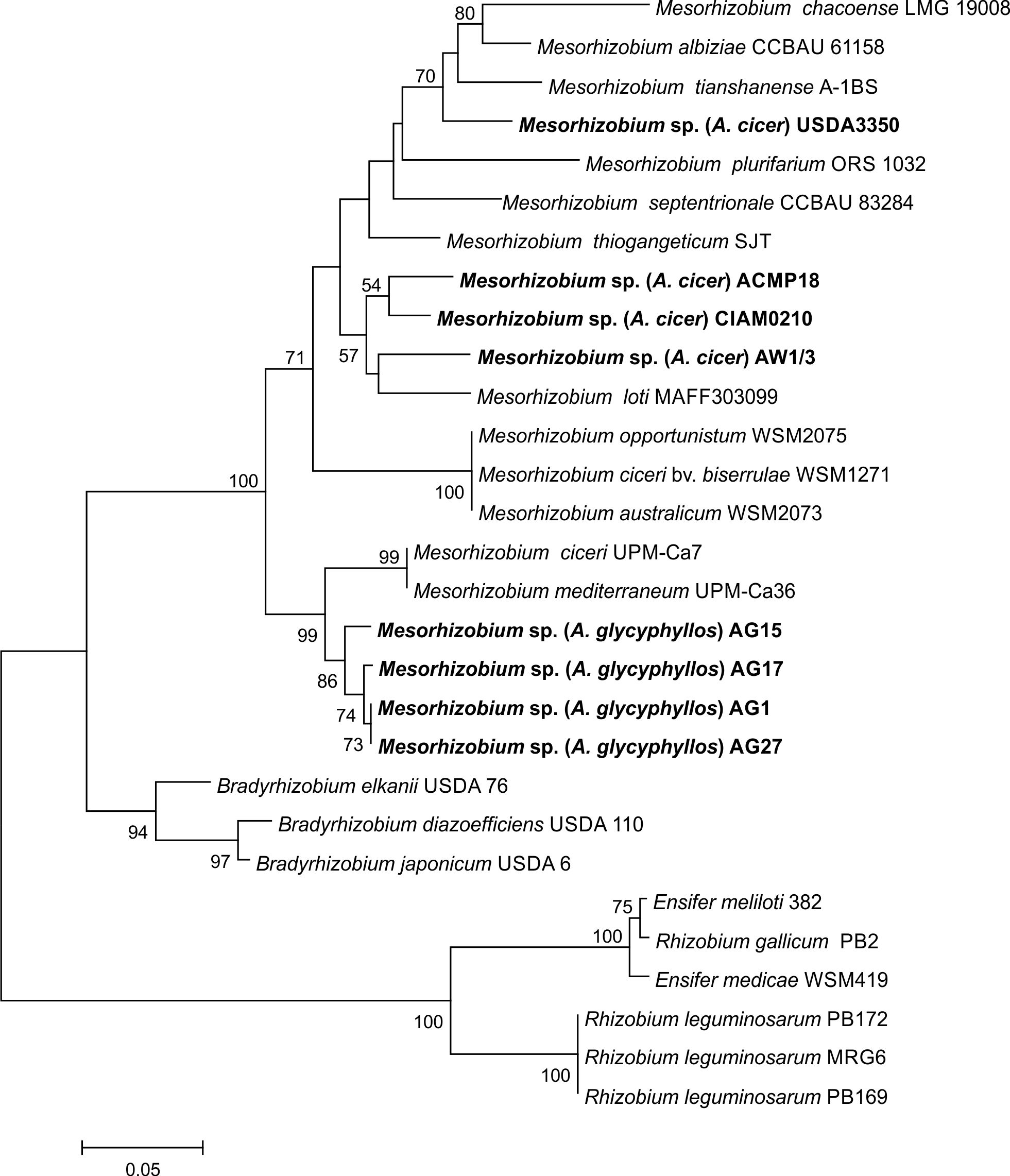

Supplement: Supplementary file 1 — Phylogenetic relationship between A. cicer and A. glycyphyllos and reference strains based on deduced AcdS sequences. Bootstrap values (1000 replicates) are shown when higher than 50%. The scale bar represents the percentage of substitutions per site (JPEG 385 kb) [file 203_2016_1243_MOESM1_ESM.jpg]
